# Quality of Life and Work Productivity Impairment among Psoriasis Patients: Findings from the National Psoriasis Foundation Survey Data 2003–2011

**DOI:** 10.1371/journal.pone.0052935

**Published:** 2012-12-28

**Authors:** April W. Armstrong, Clayton Schupp, Julie Wu, Bruce Bebo

**Affiliations:** 1 Department of Dermatology, University of California Davis, Sacramento, California, United States of America; 2 National Psoriasis Foundation, Portland, Oregon, United States of America; MGH, MMS, United States of America

## Abstract

**Objective:**

To ascertain impairment in quality of life and work productivity among patients with psoriasis and psoriatic arthritis.

**Design:**

From 2003 through 2011, the National Psoriasis Foundation collected survey data from patients with psoriasis and psoriatic arthritis via email and telephone correspondences.

**Setting:**

Survey data were collected from psoriasis and psoriatic arthritis patients in the general community in the U.S.

**Main Outcome Measures:**

Quality of life focusing on emotional impact (anger, frustration, helplessness, etc.) and physical impact (pain, pruritus, physical irritation, etc.); employment status.

**Patients:**

The surveys were performed through random sampling of participants from a database of over 75,000 patients.

**Results:**

From 2003 to 2011, 5,604 patients completed the surveys. Psoriasis and psoriatic arthritis affected overall emotional wellbeing in 88% of patients, and they interfered with enjoyment of life in 82%. Most patients reported experiencing anger (89%), frustration (89%), helplessness (87%), embarrassment (87%), and self-consciousness (89%). Many patients also actively concealed physical manifestations of their diseases (83%), and experienced pain (83%) and pruritus (93%) regularly. Of note, 12% of patients were unemployed, and 11% worked part-time. Among unemployed patients, 92% cited psoriasis and/or psoriatic arthritis as the sole reasons for not working. Among working patients, 49% missed work days regularly due to psoriasis. Compared to patients with mild psoriasis, patients with severe psoriasis have 1.8 times greater odds to be unemployed after adjusting for age and gender (Adjusted OR = 1.7, 95% CI 1.4–2.3).

**Conclusion:**

Patients with psoriasis and psoriatic arthritis continue to experience significant impairment of quality of life and work productivity.

## Introduction

Psoriasis is a chronic inflammatory disease that affects the skin, nails, and the joints. In addition to the physical impact, psoriasis can have a profound impact on patients’ psychosocial wellbeing. [1] Previous studies have shown that, regardless of psoriasis severity, nearly 60% of the patients consider psoriasis to have a major effect on their quality of life. [1] Many patients with psoriasis experience self-consciousness, embarrassment, depression, and they suffer from social isolation and stigmatization. [2], [3] Furthermore, patients with severe psoriasis were found to have significant impairment of work productivity [4], [5].

With the advent of new therapies for moderate-to-severe psoriasis in the past decade, it is important to obtain an updated assessment of quality of life among psoriasis patients. The quality of life assessments allow clinicians to determine the impact of psoriasis beyond its physical burden, and examining the effect of psoriasis on employment reveals the economic cost of having psoriasis. These data will generate the necessary evidence for continued advocacy to obtain federal and private support for psoriasis research.

The National Psoriasis Foundation (NPF) conducts semiannual surveys and collects information regarding comorbid conditions, medication use, treatment patterns, impact on quality of life, and disease burden. With over 75,000 patient members, the NPF survey enables direct assessment of quality of life from its large patient membership. This type of information is generally unavailable through chart reviews or automated extraction of data from population-based databases. Furthermore, the NPF survey allows for assessment of psoriasis severity based on self-reported body surface area involvement, which represents a direct assessment of psoriasis severity. The objectives of this study are to (1) determine quality of life among psoriasis patients focusing on emotional and physical consequences, and (2) examine work productivity impairment among psoriasis patients.

## Methods

### Study Design and Subjects

This study was approved by the Institutional Review Board at University of California Davis. Written participant consent was waived since only de-identified information was analyzed and aggregate data were presented. The NPF collects data from patients with psoriasis and psoriatic arthritis (PsA) through semiannual surveys. We used data from 13 cycles of surveys (2003–2009, 2011), which were collected from a database consisting of more than 75,000 patients. During each survey cycle, over 400 participants were identified through random sampling. Participants completed the surveys through telephone and email correspondences. Due to the large size of the database, the possibility of re-sampling of the same individuals is small, estimated at approximately 1%.

The combined survey cycles yielded 5,604 participants who completed the surveys. The respondents were asked the emotional and physical impact of psoriasis and PsA in both their personal and work lives. Many questions that inquired individual components of the emotional and physical impact (such as embarrassment) were assessed on a Likert scale that was anchored at 0 = “not at all”, 5 = ”somewhat”, and 10 = “very much”. [6] While not all questions were asked in all cycles of the survey, a substantial proportion of the survey was consistent across all survey cycles. Psoriasis severity at the time of survey was assessed by self-reported body surface area involvement (BSA). Participants’ own palm represented 1% BSA, and the participants were asked to estimate percent BSA that is affected by psoriasis. Consistent with previous NPF studies, psoriasis severity was coded mild (<3% BSA), moderate (3–10% BSA) and severe (>10% BSA). [4], [7], [8].

### Statistical Analysis

Descriptive frequency tables were calculated for all categorical variables assessing physical and emotional impact. The unadjusted association between work status and disease severity (primary independent variable) was assessed using univariate logistic regression. Multivariate logistic regression models were then used to assess this relationship adjusting for age and gender. All statistical analyses were performed using SAS 9.3. (Cary, North Carolina).

## Results

The demographic information of the NPF surveyed population is summarized in **Table 1**. The overall emotional and physical impact of psoriasis was assessed. The patients were asked regarding their feelings and attitudes towards psoriasis and PsA. Among the responders, 37.1% reported that psoriasis or PsA was a part of their identity; 25.7% reported that psoriasis or PsA was an annoyance; 20.3% reported that psoriasis caused social embarrassment, and 16.6% reported that psoriasis was physically painful. The patients were asked to assess how much of a problem psoriasis has been in their daily life on a scale of 0–10 (0 = no problem and 10 = a very large problem). A total of 5452 patients reported a median of 5 (interquartile range 3–7) (**Table 2**). Among these respondents, patients with mild psoriasis reported a median of 3 (interquartile range 1–6), whereas patients with severe psoriasis reported a median of 7 (interquartile range 4–9).

**Table 1:** Demographics of National Psoriasis Foundation Survey Participants.

	Mild Psoriasis(N = 1286)	Moderate Psoriasis(N = 2031)	Severe Psoriasis(N = 1894)	P-Value
**Sex**				0.7157
Male	498 (39%)	814 (40%)	756 (40%)	
Female	788 (61%)	1217 (60%)	1138 (60%)	
**Age**	51.4 (15.8)	49.9 (15.3)	49.9 (14.6)	0.0077
**Race**				0.0006
White	1130 (88%)	1792 (88%)	1609 (85%)	
African American	17 (1%)	27 (1%)	56 (3%)	
Asian American	20 (2%)	35 (2%)	49 (3%)	
Hispanic	51 (4%)	58 (3%)	79 (4%)	
Native American	13 (1%)	21 (1%)	25 (1%)	
Unknown	54 (4%)	92 (5%)	71 (4%)	
**BMI**	28.0 (8.6)	28.2 (6.2)	29.8 (7.4)	<0.0001
**Psoriatic Arthritis**	359 (27.9%)	637 (31.4%)	879 (46.4%)	<0.0001

**Table 2:** Mean and Interquartile Ranges for Components of Emotional and Physical Impact of Psoriasis (where 0 = not at all, 5 = somewhat, and 10 = very much).

	All Respondents	Mild Psoriasis (<3% BSA)	Severe Psoriasis (>10% BSA)
	Median (25%–75% Interquartile Ranges)		
***Emotional Impact***			
Overall emotional well being	5 (2–8)	3 (1–6)	7 (3–10)
Capacity to enjoy life	5 (1–7)	2 (0–5)	6 (3–9)
Anger or frustration	7 (3–10)	5 (1–8)	8 (4–10)
Self-consciousness	6 (3–10)	4 (0–6)	7 (4–10)
Concealment with clothing	6 (2–10)	1 (0–6)	8 (5–10)
Helplessness	6 (3–9)	5 (1–8)	7 (3–10)
Unsightly appearance	5 (2–8)	2 (1–5)	7 (4–10)
Embarrassment	5 (2–10)	2 (0–6)	7 (3–10)
Disfigurement	4 (1–7)	1 (0–4)	6 (3–9)
***Physical Impact***			
Itchiness	6 (3–9)	5 (1–8)	8 (5–10)
Physical irritation	6 (3–8)	5 (1–8)	7 (5–10)
Physical pain or soreness	5 (2–8)	4 (1–7)	7 (5–10)

**Figure 1:**
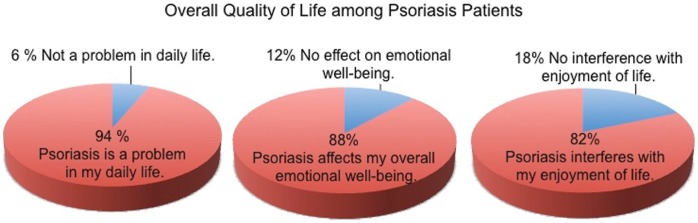
Overall Quality of Life among Psoriasis Patients.

The survey sought information regarding the overall emotional burden as well as various components of the emotional impact of psoriasis on patients. Overall, 88% of patients reported that psoriasis affects their overall emotional wellbeing, and 82% reported that psoriasis interferes with their enjoyment of life (**Figure 1**).

**Figure 2:**
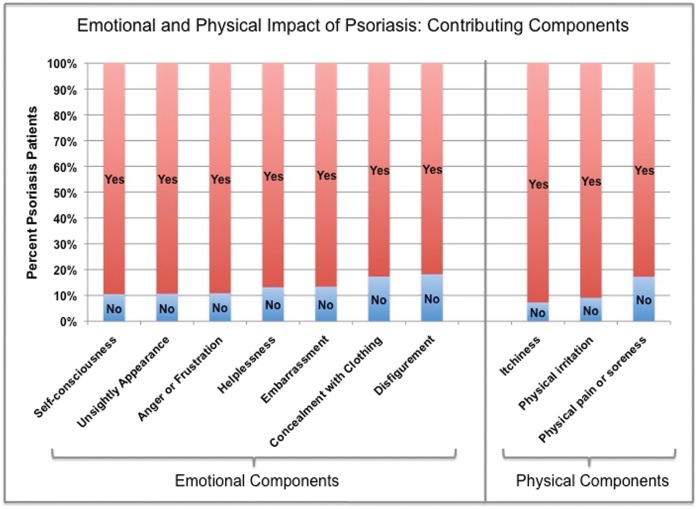
Emotional and Physical Impact of Psoriasis: Contributing Components.

On a scale of 0 to 10 (0 = not at all, 5 = somewhat, 10 = very much), the patients were asked how psoriasis has impacted the various components of their emotional wellbeing (summary results in **Figure 2**). When asked how self-conscious they feel about psoriasis, 5445 patients reported a median of 6 (interquartile range 3–10) (**Table 2**). When queried on the extent to which psoriasis makes their appearance unsightly, 5402 patients reported a median of 5 (interquartile range 2–8). When asked how angry or frustrated they feel about their psoriasis, 5447 patients reported a median of 7 (interquartile range 3–10). When asked how helpless they feel about their psoriasis, 5416 patients reported a median of 6 (interquartile range 3–9). When inquired how embarrassed they feel about their psoriasis, 5452 patients reported a median of 5 (interquartile range 2–10). When asked the extent to which clothing choices were influenced by the need to conceal their psoriasis, 5415 patients reported a median of 6 (interquartile range 2–10). When patients were asked how disfiguring their psoriasis is on a scale of 0 to 10, 5382 patients reported a median of 4 (interquartile range 1–7). For all aspects of emotional wellbeing, the patients with severe psoriasis reported greater emotional impact than patients with mild psoriasis (**Table 2**).

The number of times that psoriasis came up in conversations was also assessed in the NPF patient population. The patients reported that, on an average month, their psoriasis came up in conversations with others a mean of 5.8 times (SD±10). Among these conversations on psoriasis, 51% contained negative comments from others regarding the disease.

The physical impact of psoriasis was assessed on a scale of 0 through 10 (0 = not at all, 5 = somewhat, and 10 = very much). When asked whether their psoriasis caused itching, 5452 patients reported a median of 6 (interquartile range 3–9). When asked whether their psoriasis caused physical irritation, 5408 patients reported a median of 6 (interquartile range 3–8). When inquired whether psoriasis caused physical pain or soreness, 5442 patients reported a median of 5 (interquartile range 2–8). The dichotomized results are presented in **Figure 2**. The physical impact of psoriasis was also evaluated with respect to psoriasis disease severity. For all physical components, patients with severe psoriasis reported higher medians (7–8) than patients with mild psoriasis (4–5) (Table 2).

**Figure 3:**
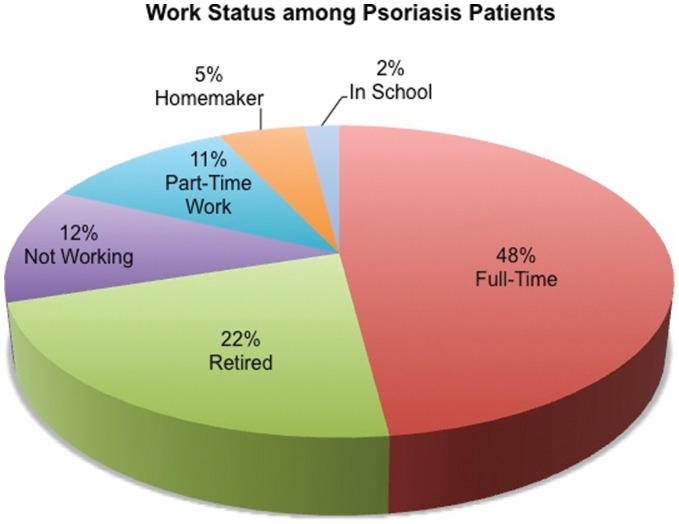
Employment Status among Psoriasis Patients.

**Figure 4:**
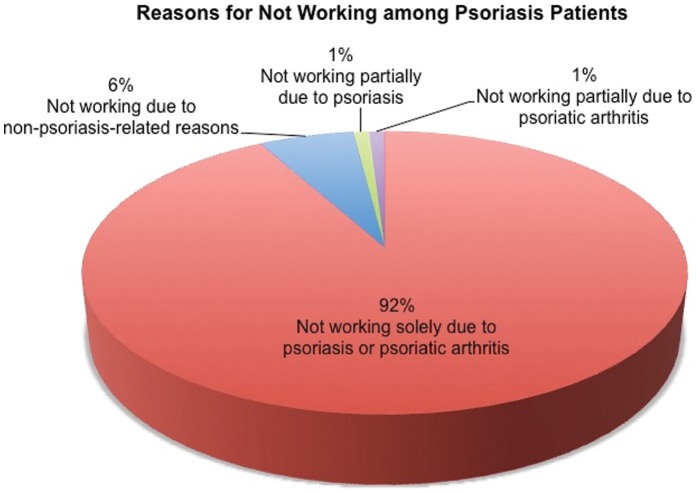
Reasons for Not Working among Psoriasis Patients.

The psoriasis patients were asked about their employment status. Less than half (48%) of the respondents were working full-time; 22% were retired, 12% were unemployed, 11% worked part-time, 5% were homemakers, and 2% were in school (**Figure 3**). Among respondents that were not working, 92% cited psoriasis and/or PsA as the sole reasons for not working (**Figure 4**). Among respondents that were working, 49% reported missing work days due to psoriasis or PsA treatment. In a typical month, among those who missed work days due to psoriasis or PsA, 62% missed 5 days or less, 6.6% missed 6–10 days, and 31% missed more than 10 days.

We also examined the association between unemployment and psoriasis severity. Compared to patients with mild psoriasis, patients with severe psoriasis have 1.8 times greater odds to be unemployed after adjusting for age and gender (AOR = 1.7, 95%CI 1.4–2.3) (**Table 3**).

**Table 3:** Unemployment and Psoriasis Severity.

	Unadjusted Odds Ratio	95% Confidence Interval
Mild Psoriasis	1	–
Moderate Psoriasis	0.99	0.77–1.26
Severe Psoriasis	1.75	1.38–2.21
	**Adjusted Odds Ratio**	**95% Confidence Interval**
Mild Psoriasis	1	–
Moderate Psoriasis	0.99	0.77–1.28
Severe Psoriasis	1.78	1.40–2.26
Age	1.03	1.02–1.04
Gender		
Male	1	–
Female	2.33	1.93–2.83

## Discussion

This study represents one of the few national ongoing efforts to regularly assess quality of life and work status impairment among psoriasis patients. The advantages of the NPF survey are several. First, the NPF survey enables detailed evaluation of the effect of psoriasis on quality of life, which is typically not feasible through chart review or data extraction from large population-based databases. Second, the large number of respondents and the sampling method allow for a representative assessment of these questions across the varying psoriasis severity cohorts. Lastly, the respondents are asked to report their psoriasis severity by body surface area involvement directly, which allows us to determine the extent to which psoriasis severity affects life quality and employment status.

While most previous studies examining quality of life in psoriasis patients were conducted in treatment-specific cohorts,[9]–[13] this study investigated quality of life among a large, representative psoriasis cohort. In this study, we found that the emotional toll of psoriasis and psoriatic arthritis on patients remains high. Specifically, greater than 80% of the respondents reported that psoriasis affects their overall emotional state and interferes with their enjoyment of life. Component scores examining the emotional impact of psoriasis revealed that it leads to a high level of anger and frustration among psoriasis patients. Patients have reported that they actively concealed their psoriatic lesions with clothing because they felt self-conscious and helpless about their psoriasis. In addition, the majority of patients experience pain and pruritus associated with their psoriasis on a regular basis. Therefore, it appears that, despite recent advances in treatments, psoriasis and psoriatic arthritis continue to have a significant emotional and physical impact on an overwhelming majority of patients. For some patients, useful ways of coping with decreased quality of life due to psoriasis may include sharing their experiences with other psoriasis patients and seeking either in-person or online support groups. [5], [6], [14] Other patients who suffer from more mental health consequences of psoriasis may need to seek professional, psychiatric help to cope with this disease.

This study reveals that psoriasis and psoriatic arthritis have considerable and deleterious economic consequences on patients. In this study, among patients who were unemployed at the time of the survey, 92% were not working due solely to psoriasis or psoriatic arthritis. Among working psoriasis patients, nearly half of them regularly missed work due either to their psoriasis or treatments related to psoriasis. These data regarding work status impairment provide further impetus for designing treatments and regimens that are flexible and patient-centered such that patients can remain productive in the workforce. [11] Long-term follow-up studies that track patients’ employment status based on their psoriasis severity and treatment regimen are necessary to determine factors influencing changes in employment status in these patients.

The study findings need to be interpreted in the context of the study design. The NPF surveys are cross-sectional such that different individuals are sampled with each survey cycle; thus, follow-up data are not available at an individual-respondent level. Furthermore, the questions used to assess quality of life were different from those used in traditional health-related quality of life instruments (such as DLQI and EuroQoL), thereby making direct comparisons to other studies difficult.[15]–[17] Finally, survey methodology is subject to self-selection bias. Specifically, differences may exist in patient characteristics between the NPF survey respondents and the general psoriasis population in the U.S. The NPF respondents might be more aware of quality of life and work productivity impairments compared to other psoriasis patients. Future studies can focus on following a large, representative cohort of psoriasis patients longitudinally to determine the effect of treatments and access to treatments on patients’ quality of life and work productivity.

In conclusion, this study presented findings from a significant, national effort to assess the impact of psoriasis on patients’ work status and emotional and physical wellbeing in the United States. Despite of recent advances in treatments, the majority of patients with psoriasis and psoriatic arthritis continue to experience significant impairment of quality of life and work productivity.
